# The emerging role of cardiovascular risk factor-induced mitochondrial dysfunction in atherogenesis

**DOI:** 10.1186/1423-0127-16-112

**Published:** 2009-12-09

**Authors:** Paolo Puddu, Giovanni M Puddu, Eleonora Cravero, Susanna De Pascalis, Antonio Muscari

**Affiliations:** 1 Department of Internal Medicine, Aging and Nephrological Diseases, University of Bologna and S. Orsola-Malpighi Hospital, Bologna, Italy

## Abstract

An important role in atherogenesis is played by oxidative stress, which may be induced by common risk factors. Mitochondria are both sources and targets of reactive oxygen species, and there is growing evidence that mitochondrial dysfunction may be a relevant intermediate mechanism by which cardiovascular risk factors lead to the formation of vascular lesions. Mitochondrial DNA is probably the most sensitive cellular target of reactive oxygen species. Damage to mitochondrial DNA correlates with the extent of atherosclerosis. Several cardiovascular risk factors are demonstrated causes of mitochondrial damage. Oxidized low density lipoprotein and hyperglycemia may induce the production of reactive oxygen species in mitochondria of macrophages and endothelial cells. Conversely, reactive oxygen species may favor the development of type 2 diabetes mellitus, mainly through the induction of insulin resistance. Similarly - in addition to being a cause of endothelial dysfunction, reactive oxygen species and subsequent mitochondrial dysfunction - hypertension may develop in the presence of mitochondrial DNA mutations. Finally, other risk factors, such as aging, hyperhomocysteinemia and cigarette smoking, are also associated with mitochondrial damage and an increased production of free radicals. So far clinical studies have been unable to demonstrate that antioxidants have any effect on human atherogenesis. Mitochondrial targeted antioxidants might provide more significant results.

## Introduction

There is a wide consensus that atherosclerosis (ATS) is an inflammatory disease associated with lipid and protein oxidation in the vascular wall [1-5]. At sites of inflammation, the local cellular environment is enriched with cytokines, chemoactractant chemokines and reactive oxygen species (ROS), such as superoxide anion, mainly produced by the activated leukocytes adhering to the endothelium. Excess ROS and reactive nitrogen species (RNS) generation has been associated with vascular lesion formation and functional defects [6-8]. ROS and RNS free radicals are molecules or molecular fragments containing one or more unpaired electrons in atomic or molecular orbitals. The unpaired electrons give the radicals a high degree of reactivity.

ROS, as well as RNS, are products of normal cellular metabolism. When there is an overproduction of ROS/RNS or a deficiency of enzymatic or non-enzymatic antioxidants, a biological damage to cellular lipids (lipoperoxidation), proteins, glucides and DNA may occur. Moreover, nitric oxide (NO) levels are reduced, due to both decreased production and increased consumption, with possible endothelial dysfunction and vascular impairment [9]. These harmful effects are termed oxidative and nitrosative stress [10].

The toxic effects of free radicals on biomolecules lead to the accumulation of damage in various cellular locations and to the deregulation of redox-sensitive metabolic and signaling pathways, and are also believed to be involved in pathological conditions such as ATS, hypertension, inflammation, diabetes, cancer and other human pathologies. There is evidence that common risk factors for coronary artery disease are associated with increased levels of ROS [11-16].

Recent studies have focused on the role that mitochondria could play in atherogenesis. In fact, mitochondria are both important sources and targets of ROS [14,15]. The mitochondrial dysfunction theory postulates that excess release of ROS and RNS from mitochondria can contribute to the inflammatory vascular reaction leading to the development of atherosclerotic lesions [17,18]. In fact, increased mitochondrial ROS production causes endothelial dysfunction, vascular smooth muscle cell (VSMC) proliferation and apoptosis of VSMCs and macrophages, with ensuing ATS lesion progression and possible plaque rupture [18].

Common cardiovascular risk factors could be involved in this process by adversely affecting the function of endothelial mitochondria, and growing evidence supports the hypothesis that mitochondrial dysfunction may be the most important unifying mechanism explaining the atherogenic action of major cardiovascular risk factors [17-19]. This review will discuss the molecular mechanisms by which atherosclerotic risk factors could lead to mitochondrial dysfunction and subsequent vascular impairment.

### ROS and RNS production in mitochondria

ROS include free radicals (mainly superoxide anion and hydroxyl) and normal molecules (such as hydrogen peroxide [H_2_O_2_] and ozone), some of which can be interconverted enzymatically [20]. For example, superoxide is converted to H_2_O_2 _by a family of metallo enzymes such as manganese superoxide dismutase (Mn SOD) or copper/zinc superoxide dismutase (Cu/Zn SOD) [21,22]. In turn, in the presence of reduced transition metals, H_2_O_2 _is mostly transformed in water by glutathione peroxidase or peroxidredoxin III (PRX III) [23].

Superoxide anion is considered the 'primary' ROS. It may be formed in the cytosol by reduction of molecular oxygen by the NADPH oxidase and xanthine oxidase. Other cytosolic or membrane-bound sources of ROS are the uncoupled endothelial nitric oxide (NO) synthase (eNOS) and the arachidonate metabolizing enzymes lipoxygenases and cycloxygenases. However, the mitochondrial respiratory chain is the major source of ROS in most mammalian cells [24-26].

Superoxide anion production can occur at complex I and III in mitochondria [27,28]. Several factors can regulate mitochondrial ROS generation. Zhang and Gutterman [24] have recently reviewed the main molecular pathways of ROS production, focusing on the effects of mitochondrial membrane potential, intracellular Ca2+, electrophilic lipids, and NO.

Nitrosative stress occurs when the generation of RNS in a biological system exceeds its ability to neutralize them [29,30]. Nitric oxide (NO) is a reactive radical acting as an important signaling molecule in several physiological processes. NO and superoxide can react together to produce peroxynitrite anion, which is a potent oxidizing agent capable of causing oxidative damage to biomolecules with subsequent inhibition of their biological function [10].

Mitochondrial ROS and RNS, as well as their metabolic products such as oxidized lipids, can also play a role in signal transduction through specific modifications of cell signaling proteins [31-33]. ROS generation in mitochondria is influenced by multiple factors, including the efficiency of the electron transport chain, oxygen concentration, the availability of electron donors such as NADH and FADH2, the activity of UCPs and cytokines, the activity of antioxidant defenses and the modulation of nuclear factors [17,34,35]. In this respect, a novel area of medical research is now developing.

### The role of uncoupling proteins (UCPs) in regulating ROS production

The mitochondrial respiratory chain requires the expression of gene products encoded by both the nuclear and mitochondrial genome [36]. Human mitochondrial genome consists of 37 genes coding for 13 proteins that function as subunits for the respiratory complexes I, III, IV, and V, whereas the genes coding for complex II are entirely nuclear. Nuclear genes play a major role in the biosynthesis of the respiratory chain and the expression of mitochondrial DNA, and all regulatory factors directing the expression of both nuclear and mitochondrial respiratory genes are of nuclear origin [36].

UCPs are mitochondrial anion transporters present in the inner mitochondrial membrane, and their role in the control of energy conversion in mitochondria has recently been demonstrated. The activation of these anion transporters allows protons to leak back into the mitochondrial matrix, thus decreasing mitochondrial membrane potential and ROS generation [37,38]. UCP2 overexpression inhibits ROS production and apoptosis induced by linoleic acid and lysophosphatidylcholine [39]. Superoxide activates UCPs, with subsequent down-regulation of its own production [40,41].

The transcriptional regulation of UCP genes, particularly UCP3 genes, is mediated to a large extent by peroxisome proliferator activated receptors (PPARs) both in normal conditions and in metabolic diseases such as diabetes or obesity [42]. PPARs, as well as liver × receptors, are nuclear receptors significantly involved in the control of lipid metabolism, inflammation, insulin sensitivity and, probably, ATS progression [1,43]. Moreover, PPARs regulate the transcription of mitochondrial and microsomal enzymes [44]. Nunn et al [45] have recently reviewed the involvement of PPARs in modulating mitochondrial proton uncoupling and ROS production.

### Mitochondrial oxidative dysfunction

Damage to mitochondria is caused primarily by the ROS generated by mitochondria themselves [34,46], mainly due to the release of electrons by the coenzymes NADH and FADH into the electron transport chain. In addition, a significant amount of ROS can be produced by the enzymes alpha-ketoglutarate dehydrogenase and monoamine oxidase located in the outer membrane of mitochondria [47,48].

The deleterious effects resulting from the formation of ROS in mitochondria are prevented to a large extent by various antioxidant systems. Under normal conditions there is a balance between ROS formation and antioxidants. However, when the antioxidant defenses become insufficient and cannot convert ROS to H_2_O_2 _fast enough, oxidative damage occurs and accumulates in the mitochondria [49]. Interestingly, free fatty acids (FFA) can decrease the mitochondrial generation of ROS under conditions of reverse electron transport, due to their uncoupling action. However, under conditions of forward electron transport, FFA increase ROS production [50].

Although somewhat controversial [51], an NO production does seem to occur in mitochondria through different pathways [52-55]. The NO produced in mitochondria [52,53] counteracts superoxide at multiple levels. It can rapidly scavenge superoxide via direct radical-radical reaction to form peroxynitrite, a potent oxidant [56-60] capable of decreasing the activity of complex I by forming S-nitrosothiols [61]. This in turn reduces mitochondrial ROS generation. Moreover, NO can facilitate superoxide scavenging indirectly by stabilizing cytochrome C and preventing its leakage from mitochondria [24].

Asymmetrical dimethyl L-arginine (ADMA), an endogenous NO synthase inhibitor [62], may lead to increased mitochondrial ROS levels [63]. Mitochondrial ROS production can also be increased by the mitochondrial *p66Shc *protein, which also favors cytochrome C release, the dissipation of mitochondrial transmembrane potential and apoptosis [64-67].

### Mitochondrial DNA oxidative damage

Mitochondrial DNA (mtDNA) is probably the most sensitive cellular target of ROS since it is located close to the inner mitochondrial membrane, where ROS are produced. Moreover, mtDNA is small in size and is not protected by histone proteins as is the case for nuclear DNA [68,69]. Many different types of oxidative DNA lesions have been described, ranging from base or sugar adduct modifications to single and double-strand breaks [70]. The hydroxyl radical can remove protons from deoxyribose, thus producing a sugar radical and inducing strand breaks and release of the affected DNA base [71]. Moreover, the hydroxyl radical can also abstract a proton from the methyl group of thymine and add it to the C4, C5 and C8 position of purines, thereby generating hydroxy adduct radicals [72].

mtDNA damage correlates with the extent of ATS [16], suggesting that mitochondrial dysfunction may promote atherogenesis [1,18,19]. Many of the DNA modifications can contribute to aging, cancer and neurodegenerative diseases [73], as well as to several other pathophysiological conditions [74]. Damage to mtDNA can have a greater impact on cellular function than damage to nuclear DNA [75].

The accumulation of mtDNA mutations can cause cell dysfunction by altering oxidative phosphorylation and Ca2+ homeostasis, inducing further oxidative stress and a defective turnover of mitochondrial proteins, and affecting the susceptibility to apoptosis [49]. In particular, the mtDNA encoded respiratory enzymes increase electron leak and ROS production, with subsequent enhanced oxidative stress and further damage to mitochondria [76]. A vicious cycle may therefore be generated, leading to progressive accumulation of ROS and oxidative damage to mtDNA [19].

### Dyslipidemia

Both cholesterol and oxidized low density lipoprotein (oxLDL) can cause mitochondrial damage [17]. Cholesterol feeding in rabbits is associated with impaired mitochondrial function [77]. Similarly, hypercholesterolemia induced mtDNA damage in heart homogenates [78]. Free cholesterol (FC) loading of macrophages [79] is associated with mitochondrial dysfunction, as suggested by decrease in mitochondrial transmembrane potential and activation of the mitochondrial apoptosis pathway, a process playing a key role in atherogenesis. In addition to the involvement of the classic apoptotic Fas pathway, in FC-loaded macrophages there is evidence of mitochondrial cytocrome C release, caspase 9 activation and increased levels of the proapoptotic protein Bax [80].

The role of redox regulation and lipid rafts in macrophages during ox-LDL-mediated foam cell formation has recently been reviewed [81]. Circulating ox-LDL represents an independent risk factor for ATS and acute cardiovascular diseases. Ox-LDL causes the mitochondrial production of ROS in endothelial cells, a process associated with apoptosis [82,83], through the activation of mitochondrial complex II [84], uncoupled eNOS, and the NADPH oxidases [85]. The exposure of endothelial progenitor or mature cells to ox-LDL results in an increased production of mitochondria-derived superoxide, with associated increase in p53 expression and subsequent activation of Bax. The activated Bax translocates into the mitochondria to release cytochrome C, which elicits the apoptotic reaction. Bax is a member of multidomain Bcl-2 proapoptotic proteins, and its activation and translocation into the mitochondria has been shown to cause mitochondrial dysfunction and cell apoptosis [86]. Cheng et al [84] have demonstrated that superoxide anion, but not hydrogen peroxide, can activate p53 and Bax. The superoxide regulation of Bax does not consist in an increase in Bax expression, but rather in an activation through a conformational change.

Vindis et al. [87] have recently shown the involvement of two distinct calcium-dependent mechanisms in ox-LDL-induced apoptosis: the first is mediated by calpain/mitochondrial permeability transitionpore/cytochrome C/caspase, while the second is mediated by a mitochondrial apoptosis inducing factor, which is cyclosporin-insensitive and caspase-independent. Ox-LDL induces apoptosis in all cells involved in ATS: endothelial cells, VSMCs, macrophages, and T lymphocytes [88,89].

### Diabetes

Type 2 diabetes mellitus (T2DM) is a multifactorial, heterogeneous, polygenic disease that accounts for > = 90% of all types of diabetes. In T2DM insulin resistance is the major pathologic feature, which often causes a compensatory increase of insulin secretion [90]. ROS are now considered a major factor in the onset and development of T2DM. ROS can induce inactivation of the signaling pathway between the insulin receptor and the glucose transporter system, leading to insulin resistance [91]. On the other hand, in addition to being a possible effect of ROS production, T2DM is also a cause of oxidative stress, with ensuing atherogenic effect [92]. Hyperglycemia induces superoxide generation in endothelial cells, and most of this superoxide may be produced by mitochondria [93]. In diabetes, electron transfer and oxidative phosphorylation are uncoupled, resulting in superoxide formation and inefficient ATP synthesis [23]. Prevention of oxidative damage represents a therapeutic strategy in diabetes [23].

In T2DM the elevation of free fatty acid (FFA) concentrations, with subsequent intramyocellular lipid accumulation, has been proposed as a cause of further insulin resistance and also pancreatic beta-cell death [94,95]. It has been reported [96,97] that both glucose and FFAs may initiate the formation of ROS via mitochondrial and NADPH oxidase mechanisms in muscles, adipocytes, beta cells and other cells. FFAs penetrate cellular organelles including mitochondria, where high ROS levels will result in lipid peroxidation and mitochondrial injury [98].

Interestingly, recent studies revealed that T2DM and insulin resistance are associated with a decreased mitochondrial oxidation function in skeletal muscle [99]. Moreover, in T2DM mitochondria are smaller, round and prone to produce superoxide [100]. Disorders of the mitochondrial transport chain, overgeneration of ROS and lipoperoxides or impairments in antioxidant defenses have been reported in T2DM, as well as in obesity.

### Hypertension

Like other risk factor for ATS, human hypertension is a condition associated with endothelial dysfunction and oxidative stress [101-107]. Mitochondrial dysfunction has been potentially implicated in both human and experimental hypertension [108,109]. Deterioration of mitochondrial energy production plays a role in the pathogenesis of hypertension in both spontaneously hypertensive rats (SHRs) [110,111] and mice [112]. Mitochondrial energy deficiency [113] and a decreased activity of complex IV have been observed in the hypertrophied myocardium from SHRs [114]. In these animals there is also an abnormal transport of inorganic phosphate in left ventricular mitochondria [115]. Overall, these data indicate that some defect in the regulation of mitochondrial ATP synthase activity occurs in the cardiomyocites of SHRs. In addition, mitochondrial calcium overload could significantly contribute to the development of hypertensive states [113].

An association of hypertension with mitochondrial uncoupling proteins (UCPs) has been reported both in experimental and human hypertension. In particular, mice with doxycycline-inducible expression of UCP 1 in arterial walls develop hypertension and dietary ATS [112]. A common polymorphism of the UCP2 gene was associated with hypertension in a Japanese population, and with hypertension and obesity in Caucasians [116].

ROS and RNS can damage mtDNA [117], with decreased energy production, additional generation of ROS, and enhancement of the cellular signals capable of initiating hypertension, as well as ATS [117]. mtDNA mutations have been demonstrated in black Americans with hypertension-associated end-stage renal disease [118]. Moreover, it has been shown that a mutation in mitochondrial tRNA is associated with hypertension, hypercholesterolemia and hypomagnesemia [119]. The putative role of mitochondrial dysfunction in hypertension has been recently reviewed [120].

### Aging, hyperhomocysteinemia, cigarette smoking and HIV as risk factors

Aging significantly increases the risk of coronary heart disease and other vascular diseases. Several human and animal studies have shown an age-related impairment of mitochondrial respiratory chain function and ATP synthesis, together with an accumulation of oxidative mtDNA mutations [18]. It has been suggested that mitochondrial dysfunction is a major contributor to aging and aging-associated ATS [18].

Elevated plasma homocysteine is an independent risk factor for ATS. It has been proposed that endothelial dysfunction and ATS can be induced by homocysteine through increased generation of ROS and reduced NO availability [121-124]. Austin e al. [125] have shown that homocysteine promotes mitochondrial damage and alters mitochondrial gene expression and function. Further, homocysteine stimulates the expression of NRF1 and T-fam, two nuclear transcription factors involved in the modulation of mitochondrial biogenesis. This effect was prevented by pre-treatment with antioxidants, suggesting that ROS are important mediators of the effects of homocysteine. In addition, homocysteine induces endothelial cell apoptosis through mitochondrial mechanisms [126,127].

Cigarette smoking may significantly increase the risk of early ATS by affecting mitochondrial function. In fact, in addition to endothelial injury, platelet activation and LDL oxidation, the atherogenic effects of cigarette smoking include oxidative mtDNA damage with mtDNA deletions and loss of mitochondrial membrane potential [13,128-131].

Finally, mitochondrial dysfunction can also be considered a contributing factor for HIV-associated ATS [132-134].

Thus, a range of seemingly unrelated conditions has underlying pathophysiological mechanisms in common, namely ROS production and accumulation of mtDNA damage, resulting in mitochondrial dysfunction and ATS.

## Conclusion

Based on experimental evidence and clinical studies, oxidative and nitrosative stress have been shown to be induced by ATS risk factors and to contribute to the onset and development of atherosclerotic vascular damage. Moreover, endogenous risk factors, such as hypertension and diabetes, may be both the cause of vascular ROS generation and a consequence of ROS induced endothelial dysfunction.

Under physiological conditions, the mitochondrial respiratory chain is a major source of superoxide and other ROS [135-139]. This mechanism may be triggered by risk factors, with subsequent endothelial dysfunction and atherogenesis. On the other hand, mitochondria may be not only a relevant source, but also a target of ROS [14,15]. In fact, an excessive production of ROS in mitochondria will damage lipids, carbohydrates, and proteins, as well as mtDNA. Indeed, oxidative mtDNA mutations could represent an important step in the chain of events connecting risk factors to atherogenesis, acting as further causes of ROS generation [78,140]. Ballinger et al. [16] suggested that mitochondrial damage in an early stage can predict ROS and RNS-mediated atherosclerotic lesions. Overall, the pathogenetic role of mitochondrial dysfunction in atherogenesis may now be considered more than a plausible hypothesis [19].

Despite the experimental evidence of the importance of oxidative stress in inducing ATS, clinical studies have been unable to demonstrate that antioxidants have any effect on human atherogenesis [141-143]. Some studies have shown that antioxidants such as alpha tocopherol, ubiquinone and N-acetylcysteine could decrease mitochondrial oxidative damage in different experimental models [144-148]. However, the effectiveness of these compounds is limited, since they do not significantly accumulate within mitochondria [149], and strategies for the targeted delivery of antioxidants to mitochondria are now in the developmental stages [150]. In fact the antioxidant moieties can be bound by covalent attachment to lipophilic triphenylphosphonium cations, which, due to the large mitochondrial membrane potential, do accumulate within the mitochondria [151]. The targeted version of ubiquinol (MitoQ) has been used most extensively [151,152], and is now being tested in man as an oral drug for the treatment of hepatitis C [153] and Parkinson's disease [154]. However, despite these promising data, more pre-clinical and clinical studies are needed in order to evaluate both the effectiveness and the safety of mitochondria targeted antioxidants.

## Competing interests

The authors declare that they have no competing interests.

## Authors' contributions

PP designed the article and wrote the first draft. GMP specifically prepared the sections concerning mitochondrial oxidative dysfunction and mitochondrial DNA oxidative damage. EC specifically prepared the sections concerning dyslipidemia and diabetes. SDP contributed to literature search and edited the manuscript. AM participated in designing the article, revised critically the manuscript, and re-arranged the final version. All authors read and approved the final manuscript.
